# Anticancer Effects of Green Tea and the Underlying Molecular Mechanisms in Bladder Cancer

**DOI:** 10.3390/medicines5030087

**Published:** 2018-08-10

**Authors:** Yasuyoshi Miyata, Tomohiro Matsuo, Kyohei Araki, Yuichiro Nakamura, Yuji Sagara, Kojiro Ohba, Hideki Sakai

**Affiliations:** Department of Urology, Nagasaki University Graduate School of Biomedical Sciences, 852-8501 Nagasaki, Japan; tomozo1228@hotmail.com (T.M.); k-araki205@cameo.plala.or.jp (K.A.); yn1238056@yahoo.co.jp (Y.N.); gaasara3@gmail.com (Y.S.); ohba-k@nagasaki-u.ac.jp (K.O.); hsakai@nagasaki-u.ac.jp (H.S.)

**Keywords:** animal model, treatment, bladder cancer, epidemiology, green tea

## Abstract

Green tea and green tea polyphenols (GTPs) are reported to inhibit carcinogenesis and malignant behavior in several diseases. Various in vivo and in vitro studies have shown that GTPs suppress the incidence and development of bladder cancer. However, at present, opinions concerning the anticancer effects and preventive role of green tea are conflicting. In addition, the detailed molecular mechanisms underlying the anticancer effects of green tea in bladder cancer remain unclear, as these effects are regulated by several cancer-related factors. A detailed understanding of the pathological roles and regulatory mechanisms at the molecular level is necessary for advancing treatment strategies based on green tea consumption for patients with bladder cancer. In this review, we discuss the anticancer effects of GTPs on the basis of data presented in in vitro studies in bladder cancer cell lines and in vivo studies using animal models, as well as new treatment strategies for patients with bladder cancer, based on green tea consumption. Finally, on the basis of the accumulated data and the main findings, we discuss the potential usefulness of green tea as an antibladder cancer agent and the future direction of green tea-based treatment strategies for these patients.

## 1. Introduction

Green tea is obtained from the leaves of the plant *Camellia sinensis*, and it is one of the most popular beverages worldwide, particularly in Asia. Green tea is recognized to have health-promoting effects that are attributed to green tea polyphenols (GTPs), such as epicatechin, epigallocatechin, epicatechin-3-gallate (ECG), and epigallocatechin-3-gallate (EGCG) [1]. In addition, its antioxidant, anti-inflammatory, and antibacterial properties have been reported to provide various benefits to its consumers [2]. Several investigators have reported that the addition of GTPs to drinking water can prevent carcinogenesis and the development of various types of malignancies [3,4,5]. In addition, laboratory-based evidence of the anticancer effects of GTPs has been increasing [4,6]. On the other hand, other studies have shown that green tea consumption does not decrease the risk for various types of cancers, including brain cancer and breast cancer [7,8]. In addition, a meta-analysis of published case-control and cohort studies showed that green tea consumption is not associated with risk of pancreatic cancer [9]. Furthermore, no significant association between green tea consumption and colon cancer risk was observed (summary RR: 0.99; 95% CI: 0.79, 1.24) in prospective cohort studies [10]. Additionally, it has been reported that green tea consumption might be associated with an increased risk of upper aerodigestive tract cancer [11]. Taken together, the anticancer effects of green tea consumption and GTPs remain controversial. With regard to bladder cancer, various in vivo and in vitro studies have provided inconsistent results.

Below, we first present a general discussion on the relationship between green tea consumption and cancer risk on the basis of data from epidemiological studies, the mechanisms underlying these anticancer effects, the safety and adverse effects of GTPs, and trials of treatment strategies based on green tea consumption. Then, we present a detailed review of these topics with a focus on bladder cancer. Previously, excellent reviews of studies, particularly epidemiological investigations, on the relationship between green tea consumption and bladder cancer risk have been published [12,13]. Therefore, in the present review, we paid attention to the molecular mechanisms underlying the anticancer effects, findings from studies involving animal models, and novel treatment strategies based on green tea consumption for patients with bladder cancer, as these topics have been less well addressed in previous reviews.

## 2. Anticancer Effects of Green Tea Polyphenols

GTPs exhibit anticancer effects via the regulation of different cancer-related processes and factors, including DNA methylation, histone modification, micro-RNA, and proteins [5,14,15,16,17]. They also play an important role via the regulation of apoptosis, growth, invasion, and angiogenesis in various types of malignancies [13,15,18,19]. A recent study showed that GTPs can influence the pathological roles of not only cancer cells, but also cancer stem cells [17]. These findings have led to a hypothesis that GTPs have strong anticancer effects in vivo and in vitro. In the next section, findings that support this hypothesis are discussed.

### 2.1. Anticancer Effects Shown in Epidemiological Studies

When discussing the anticancer effects of GTPs, it is necessary to focus on the different phases in the natural evolution of malignant cells. Briefly, green tea extracts are thought to be useful for primary prevention (prevention and delay of cancer onset) and tertiary prevention (prevention of recurrence and metastasis) [17]. With regard to primary prevention, several epidemiological studies have shown that the average age at cancer onset in individuals with high levels of green tea consumption was significantly higher than that in individuals with low levels of green tea consumption [17,20,21]. These studies have also shown that the consumption of more than 10 cups of green tea was associated with a reduced incidence of lung, colorectum, liver, and stomach cancer [17,20,21]. Furthermore, several reports have shown that GTPs were effective in preventing transformation from a premalignant status to frank malignancy [14,22].

With regard to tertiary cancer prevention, on the other hand, one study reported that the recurrence rate for stage I and stage II breast cancer was significantly lower for patients consuming ≥5 cups of green tea daily (16.7%) than in those consuming ≤4 cups (24.3%; *p* < 0.005) [23]. However, these preventive effects were not observed for stage III breast cancer recurrence. Another report stated that the hazard ratio for stage I breast cancer recurrence showed a statistically significant decrease (hazard ratio, 0.43; 95% confidential interval, 0.22–0.84) [24]. Similar preventive effects on cancer recurrence have also been reported for other cancers. For example, a study showed that the recurrence rate for colorectal cancer was significantly lower in an experimental group (daily consumption of green tea plus tablets of green tea extracts (GTEs) than in a control group (31.0% vs. 15.0%; *p* < 0.05) [25]. In addition, GTEs reportedly showed inhibitory effects on metastasis in various types of malignancies. For example, lung metastasis from melanoma was inhibited by GTEs in an animal experiment [26].

### 2.2. Mechanisms Underlying the Anticancer Effects of Green Tea

Various cancer-related molecules have been reported to be modulated by GTPs. In fact, many studies have reported that GTPs inhibited cell growth and induced apoptosis in various types of malignancies [19,27,28]. In addition, these polyphenols modulate the functions of various cancer-related signaling molecules. For example, the function and expression of vascular endothelial growth factor (VEGF), which is a strong stimulator of angiogenesis, are mediated by GTPs in oral cancer and esophageal squamous cell carcinoma [14,29]. Furthermore, in these malignancies, cyclin D1, which is a cell cycle regulator, and caspase-3, which is an important determinant for apoptosis, are associated with GTPs [14,29].

EGCG has also been reported to affect the pathological behavior of stem cells in several cancers. For example, they inhibited the proliferation and induced the apoptosis of lung cancer stem cells [30] and suppressed tumor spheroid formation by colorectal cancer stem cells [16].

Numerous previous reports have presented detailed information on the mechanisms underlying the general anticancer effects of green tea; thus, we present the details only for bladder cancer.

### 2.3. Cancer Treatment Strategies Based on Green Tea Consumption

While GTPs inhibit tumor growth in human lung cancer cell lines, their inhibitory effects were approximately 250-fold less effective than those of doxorubicin (Adriamycin) [31]. Therefore, it has been suggested that GTPs are more suitable for use in cancer prevention than as cancer treatment [17]. In fact, chemoprevention by oral administration of green tea catechins was investigated in volunteers with high-grade prostate intraepithelial neoplasia, a precancerous change [22]. Furthermore, EGCG administration was reported to be useful as an adjuvant therapy after complete tumor resection [32]. Various GTPs can also increase the sensitivity of anticancer agents and prevent drug resistance [33,34]. In fact, new cancer treatment strategies using a combination of GTPs and anticancer agents, such as cisplatin, paclitaxel, and tamoxifen, have been recommended for several types of malignancies [35,36]. The molecular mechanisms underlying these synergistic effects are partially clear. For example, in osteosarcoma, SOX overlapping transcript variant 7 is known to contribute to an improvement in the clinical efficacy of combination therapy with EGCG and doxorubicin via inhibition of both autophagy and stemness [37]. In addition to anticancer agents, various natural compounds have also been shown to exhibit anticancer effects when combined with GTPs. For example, in a xenograft model of human leukemia, the oral intake of green tea and intraperitoneal injection of quercetin, which is a polyphenol present in fruits and vegetables, induced apoptosis and suppressed tumor growth [38]. Furthermore, another study reported that EGCG and quercetin enhanced the anticancer effects of doxorubicin via regulation of cell cycle arrest and apoptosis in prostate cancer [39].

As mentioned above, the anticancer effects of GTPs are relatively weak, and a higher concentration of GTPs near cancer cells is considered to improve their anticancer effects. Polymeric micelles are used as the drug-delivery systems in various treatments. In recent years, several anticancer drug-loaded micellar nanocomplexes based on GTE-derivatives have been developed [40,41]. A micellar nanocomplex based on the self-assembly of EGCG and doxorubicin inhibited tumor growth in a xenograft model of human liver cancer [41]; similar results were reported for a micellar nanocomplex prepared by complexation of oligomerized EGCG with trastuzumab (Herceptin) in a mouse model of breast cancer [40]. These findings indicate that more detailed in vivo studies and clinical trials of such combined therapies are important for development of new treatment strategies of bladder cancer.

## 3. Bladder Cancer and Green Tea

Bladder cancer is a common malignancy in 43 countries around the globe, although the incidence among men, women, and both sexes has decreased in 11, 10, and 12 countries, respectively [42]. Bladder cancer imposes a substantial psychological, physical, and economic burden, and its prognosis is poor, despite various multidisciplinary therapeutic approaches, particularly in cases with muscle invasion and/or metastasis [43]. Therefore, more detailed information will be useful for devising preventive and treatment strategies.

Many studies have focused on the clinical benefits of food choices in terms of chemoprevention, treatment, and survival in bladder cancer [44]. Furthermore, complementary and alternative medicine (CAM), defined as a diverse medical and healthcare system, is recognized as a strategy commonly used by patients with malignancies, including bladder cancer [45]. Green tea consumption and GTPs intake are suggested as CAM strategies [46].

### 3.1. Epidemiological Studies on Bladder Cancer

#### 3.1.1. Case-Control and Cohort Studies

As mentioned above, no consensus has been derived from the findings of epidemiological studies on green tea consumption, and the effects thereof on carcinogenesis and tumor development and growth remain controversial. Similarly, with regard to bladder cancer, opinions about the relationship between green tea consumption and cancer risk are conflicting. For example, it was reported that the risk of bladder cancer in individuals with high green tea consumption (≥0.14 servings/day) was lower than that in individuals who never consumed the beverage (odds ratio, 0.60; 95% confidential interval, 0.45–0.79; *p* < 0.001) [47]. However, to our knowledge, this is the only study supporting the hypothesis that a high level of green tea consumption suppresses the risk of bladder cancer. On the other hand, surprisingly, another study reported that the risk of bladder cancer was significantly higher for individuals who consumed five to nine cups of green tea daily than in those who did not consume green tea (odds ratio, 2.67; 95% confidential interval, 0.49–2.84) [48]. However, the same study also showed that bladder cancer risk was similar for individuals who consumed ≥10 cups daily than for those who did not consume green tea (odds ratio, 1.18; 95% confidential interval, 1.44–4.94) [48]. Table 1 summarizes the findings of previous studies on the relationship between green tea consumption and bladder cancer risk.

As shown in Table 1, four of the seven studies were performed in Asian countries. In addition, among the three studies performed in the USA, the subjects were of Japanese ancestry in one and in another were Japanese individuals living in the USA [47,49,50]. Case-control and cohort studies have not been performed in Central and South America, Europe, or Africa. This does not mean that researchers and residents in these countries are not interested in the anticancer effects of tea extracts, including catechins, in bladder cancer. In fact, to clarify the relationship between fluid intake and the risk of urothelial cancer, a multicenter cohort study was designed for 233 and 236 subjects from 23 centers in 10 European countries [54]. The findings of that study revealed that the intake of tea and herbal tea was not associated with the risk of urothelial cancer. However, the effects of green tea per se were not investigated. Moreover, information concerning the anticancer effects of green tea in white and black individuals is scarce.

With regard to epidemiological studies, we should also pay attention to the fact that various units are used to measure green tea consumption, such as frequency/day, cups/day, and mL/day. In addition, the volume per cup differs among countries and regions. For example, the volume of representative cups used for consuming green tea in Japan (120–140 mL) is less than that in Western countries. This information is important when considering the clinical usefulness of green tea. It would be useful if more detailed information about the amount of green tea consumed were included in such reports in future.

#### 3.1.2. Meta-Analyses

There have been several reports of meta-analyses of the relationship between green tea consumption and bladder cancer risk. In this review, we have focused on meta-analyses published in English, because we could not verify the content of publications in other languages. A summary of these meta-analyses is presented in Table 2.

Only one meta-analysis showed a negative correlation between green tea consumption and bladder cancer risk [13], while the remaining four showed no association between green tea consumption and the risk. A meta-analysis of three cohort studies and three hospital-based case-control studies showed that green tea consumption was not related to a decreased risk of bladder cancer (odds ratio, 0.97; 95% confidential interval, 0.73–1.21) [55]; in addition, another meta-analysis of two cohort studies and three case-control studies showed that there was no significant relationship (relative risk, 1.03; 95% confidential interval, 0.82–1.31) between green tea consumption and bladder cancer risk [56]. Yet, another dose–response meta-analysis of 25 case-control studies and seven prospective cohort studies showed no significant association between green tea consumption and bladder cancer risk [58].

On other hand, it should be noted that the number of studies included in these meta-analyses was relatively small. Although the meta-analysis by Weng et al. assessed the relationship between the consumption of various types of tea (black tea, oolong tea, herbal tea, etc.) and bladder cancer risk in 32 studies (25 case-control studies and seven cohort studies), it included only seven studies involving green tea consumption [58]. Another limitation of these meta-analyses is that they did not include papers published in languages other than English. We found a meta-analysis of five studies showing that increased consumption of green tea may have a protective effect on bladder cancer (odds ratio, 0.76; 95% confidential interval, 0.66–0.95); however, only three of those five studies were verifiable [59]. Considering that green tea is a major beverage for Asians, the majority of whom are not native English speakers, we believe that the publication of English versions of studies published in Asian languages is important.

### 3.2. Mechanisms Underlying the Anticancer Effects of Green Tea in Bladder Cancer

Although it remains unclear how GTPs regulate malignant aggressiveness, various cancer-related mechanisms and molecules are known to play important roles in the anticancer effects of green tea. In addition, green tea and its extracts have been suggested to affect multiple pathological activities and signaling pathways [60]. Therefore, we evaluated the relationships between GTP and cancer-related functions, including cell survival, cell death, migration, and the cell cycle, in bladder cancer cell lines (Table 3).

#### 3.2.1. Cancer Cell Proliferation and Cell Death

In vitro studies have shown that EGCG suppressed cancer cell proliferation and growth in various types of bladder cancer cell lines. Interestingly, one study showed that a high concentration (60 μg/mL) of ECG and ECGC significantly suppressed the proliferation of bladder cancer cell lines (RT4, SW780, TCCSUP, and T24) [65]. However, the same study also showed that a low concentration (10 μg/mL) of ECG could suppress proliferation in SW780, but not in RT4 and T24 [65]. Thus, we speculate that the inhibitory effects of green tea polyphenols on bladder cancer cell proliferation are regulated by complex mechanisms.

In addition to its effects on cancer cell proliferation, EGCG induced apoptosis in various types of bladder cancer cell lines, regardless of the malignant potential and species (Table 3). A study showed that EGCG played an important role in not only the induction of apoptosis, but also the inhibition of growth in a bladder cancer cell line (NBT-II) [62]. However, another study showed that EGCG affected only the apoptosis of bladder cancer cells (SW780), but not cell proliferation or migration [27].

Autophagy is a highly conservative catabolic process used by eukaryotic cells for the degradation of damaged or superfluous proteins and organelles [66]. Although autophagy plays important roles in cell signaling and cellular homeostasis, including physiological cytoprotective or prosurvival mechanisms, completely uncontrolled or excessive autophagy has been associated with cell death [68]. Autophagy is also recognized as an important factor for understanding the pathological characteristics and for planning treatment strategies for many types of cancers [69,70]. This process is stimulated by various stimuli, including nutrients [71,72]. In fact, in bladder cancer cells, autophagy plays an important role in pathological processes and signaling pathways via external stimuli, including natural products [73,74]. With regard to the relationship between autophagy and GTPs, an in vitro study showed that the latter inhibited epirubicin-induced autophagy [75].

Thus, GTPs, especially EGCG, play important roles in cell survival, and affect cell proliferation and cell death in various types of bladder cancer cell lines. On the other hand, as shown in Table 3, the dosage and concentration of EGCG used in in vitro studies varied greatly. Further studies are necessary to judge the optimal dosage and concentration to ascertain the relationship between GTP and bladder cancer cell survival.

#### 3.2.2. Other Cancer-Related Mechanisms

Cancer cell migration and invasion are two key processes for cancer cell dissemination and metastasis in almost all solid tumors, including bladder cancer. As shown in Table 3, in vitro studies have used scratch assays and migration assays to show that EGCG suppressed cell migration and invasion. However, information pertaining to cell migration and invasion is scarce relative to that related to cell survival and death. On the other hand, the concentrations of EGCG that inhibited bladder cancer cell migration were typically lower than those that affected tumor growth and apoptosis (Table 3). More detailed investigations are needed to clarify this issue.

A previous study showed that EGCG caused cell cycle arrest in rat transitional cell cancer [62]. Another study suggested that tea polyphenols can deregulate the cell cycle in bladder cancer cells [46]. Oxidative stress is defined as an imbalance between the cellular production of reactive oxygen species (ROS) and antioxidants; excessive production of ROS causes DNA damage and promotes the activities of oncogenes and/or inhibits tumor suppressor genes [76]. Oxidative stress plays important roles in the aggressiveness of bladder cancer [77]. A previous study has shown that GTPs can protect against oxidative stress in the event of bladder cell death, although this effect was not detected in one of the bladder cancer cell lines (T24) [78].

On the other hand, as shown in Table 3, EGCG has been most widely used for in vitro studies aiming to clarify the relationship between GTPs and various cancer-related functions. Furthermore, other reports have shown that EGCG is the most important component in terms of the health benefits and anticancer effects of green tea [79,80]. Therefore, ECGC has been the focus of discussion in terms of both potential preventative and of therapeutic approaches for bladder cancer.

#### 3.2.3. Cancer-Related Potential Molecular Targets of Green Tea Polyphenols

Next, we evaluate reports on the relationships between GTPs and cancer-related signaling molecules in bladder cancer cell lines (Table 4).

As shown in Table 4, GTPs can regulate the expression and activity of various types of cancer-related molecules. Among them, the bcl-2 and caspase families are well-known important regulators of apoptosis in many types of cancer cells, including bladder cancer cells [81,82,83]. In addition, phosphatidylinositol 3-kinase (PI3K)/Akt signaling is one of the most important mediators for the regulation of pathological activities such as cell proliferation, apoptosis, and progression in malignancies, including bladder cancer [82,84,85]. Furthermore, a JNK/Bcl-2/Beclin-1-mediated mechanism is associated with autophagy and apoptosis in bladder cancer cells [68]. As a mechanism underlying the pro-apoptotic function, the regulation of nuclear factor-kappa B and matrix metalloproteinase (MMP)-9 has been suggested [27]. Various apoptosis-related molecules have been associated with GTP-induced cell death in bladder cancer cells. Surprisingly, however, there has been no study of the relationship between GTPs and p53 status and function in bladder cancer, even though significant mechanistic associations have been reported in other types of malignancies [86,87]. Therefore, the effects of GTPs on bladder cancer cell proliferation and death require further investigation.

MMP-9 and N-cadherin, which play crucial roles in cancer cell migration and invasion in bladder cancer [88,89], are reportedly modulated by GTPs in bladder cancer cell lines [27,63]. However, there is little information on the effect of GTPs on E-cadherin, which is the best known and classical member of the cadherin family, in bladder cancer. Similarly, the influence of GTPs on MMP-2, which is a strong stimulator of cell invasion and migration, remains unclear. However, there has been a report that a mixture of lysin, proline, arginine, ascorbic acid, and GTEs inhibited cancer cell invasion via the regulation of MMP-2 and MMP-9 production in a bladder cancer cell line (T24) [90].

In a bladder cancer cell line established from Wistar rats (NBT-II), EGCG caused growth inhibition and cell cycle arrest via the downregulation of cyclin D1, cyclin-dependent kinase 4/6, and retinoblastoma protein in terms of regulation of cell cycle progression [62]. As mentioned above, several investigators have reported that GTPs play an important role in cell cycle regulation in bladder cancer cells [46,62]. However, more detailed information on the molecular mechanisms is necessary to understand the antibladder cancer effects of green tea.

Proteomic analysis has shown that EGCG affects the expression levels of heat shock protein 27 in the TSGH-8301 cell line [66]. Interestingly, a recent study showed that heat shock protein 27 expression was significantly associated with clinicopathological features, including the stage and grade, of bladder cancer in 132 patients [91]. The same study also showed no association with clinical outcomes, such as tumor recurrence, progression, and patient survival. Detailed mechanistic links between GTPs and heat shock protein 27 in other diseases, including cancers, have been reported, and knowledge of changes in heat shock protein 27 activities by green tea are important for the evaluation of cancer-related signal pathways and development of new therapeutic strategies [92]. With regard to bladder cancer, more detailed investigations of GTP-induced anticancer effects of heat shock protein 27 are therefore necessary.

In summary, various types of cancer-related molecules are modulated by GTPs. These molecules usually play multiple roles in the malignant behavior of most cancers, including bladder cancer. For example, MMP-9 plays an important role in apoptosis and cell migration, while Wnt signaling plays an important role in cell proliferation, cell cycle regulation, apoptosis, and migration in bladder cancer [93,94]. We propose that identification of detailed molecular mechanisms of anticancer cell effects by GTPs is essential for targeting the key signaling molecules.

#### 3.2.4. Correlation with Genetic Polymorphisms

A previous report has showed that, although the frequency of green tea consumption was not significantly associated with breast cancer risk in all women or in women with the low-activity genotype of the angiotensin-converting enzyme (ACE), a significant decrease in cancer risk with an increase in the frequency of green tea consumption was found in women bearing the high-activity genotype of ACE [95]. Furthermore, the same study group showed that methylenetetrahydrofolate reductase and thymidylate synthase genotypes affected the association between green tea consumption and breast cancer risk [24]. In recent years, several studies on various malignancies have shown the relationship between the anticancer effects of GTPs and genetic polymorphisms [96,97]. With regard to bladder cancer, a study reported that genotype was significantly associated with tea consumption-associated changes in bladder cancer risk [47]. Tea consumption did not decrease the risk of bladder cancer for rs7571337 AA-genotype carriers, whereas it decreased the risk for carriers of the rs7571337 AG+GG genotypes [47]. Unfortunately, the study evaluated the effects of a mix of green tea, black tea, decaffeinated tea, and other herbal teas, rather than those of green tea alone [47]. In another study, lymph node metastasis from urothelial cancer was associated with the methylation status of *DACT1*, which is controlled by polymorphisms in the gene encoding methylenetetrahydrofolate reductase (MTHFR) [98]. Similarly, *MTHFR* 677T polymorphisms were reported to be associated with hypomethylation of insulin-like growth factor-2, the proportion of which was significantly associated with lymph node metastasis [99]. However, such DNA methylation was not significantly associated with green tea consumption [98,99].

In summary, the anticancer effects of GTPs according to genetic polymorphisms in patients with bladder cancer require further studies, with an emphasis on the prevention of carcinogenesis and tumor progression in multiple population groups. Of note, most previous studies were conducted in Asian subjects.

### 3.3. Anticancer Effects of Green Tea in Animal Models

Various in vivo studies using animal models have shown that GTEs may be useful therapeutic agents for patients with bladder cancer (Table 5).

While one study reported that GTPs inhibited tumor growth and invasion via regulation of angiogenesis in a mouse model of chemically (*N*-butyl-*N*-(4-hydroxybutyl)-nitrosamine; BBN) induced bladder cancer [102], another reported that green tea infusion did not affect the development of bladder cancer after chemical induction in a mouse model [103]. However, the latter study used whole green tea, not GTEs. In another study, GTP intake suppressed the expression of cyclooxygenase-2, hemeoxygenase-1, and human antigen-R (HuR), and this suppression of cancer-related molecules led to the inhibition of cancer cell proliferation and angiogenesis in a mouse model of BBN-induced bladder cancer [104]. Interestingly, the study also found that VEGF-A expression was not directly affected by green tea intake, although it was regulated by green tea intake via HuR regulation [104]. Therefore, green tea intake is speculated to regulate various malignant behaviors through complex mechanisms.

Some rat models of bladder cancer also showed the anticancer effects of green tea. In a study of Fisher 344 rats implanted with urothelial cancer cells (AY-27), 18 of 28 (64%) rats who received intravesical EGCG instillation were free of tumors, whereas all 12 control rats developed tumors [61]. These findings indicated that intravesical EGCG instillation significantly (*p* = 0.001) suppressed the growth of urothelial cell carcinoma in rats [61]. Another study involving a rat model of bladder cancer induced by the intake of drinking water containing BBN showed that the number and volume of tumors were significantly fewer in rats treated with powdered green tea than in control rats that received no such treatment [101]. The same authors also showed dose-dependent anticancer effects of green tea powder intake in a similar rat model of bladder cancer [100]. On the other hand, it has been reported that green tea infusion influences urothelial inflammation, but not carcinogenesis, in a mouse model of BBN-induced bladder cancer [103]. However, in that study, mice treated with BBN only showed no cancer cells.

Taken together, the anticancer effects of GTPs were confirmed in various animal models, and these results support the need for preclinical studies and clinical trials of the use of GTPs in the prevention of recurrence and progression in patients with bladder cancer.

### 3.4. Bladder Cancer Treatment and Prevention Strategies Based on Green Tea Consumption

Epirubicin is an effective anticancer agent for bladder cancer. Epirubicin reportedly induces this cytoprotective autophagy in bladder cancer cell lines [67]. Interestingly, GTPs inhibited epirubicin-induced autophagy and promoted apoptosis in the same study [67]. Accordingly, it was suggested that GTPs can be used in combination with epirubicin for enhanced epirubicin-based bladder cancer therapy.

Discrepancies in findings regarding the anticancer effects of green tea between epidemiological studies and in vitro studies can be attributed to differences in the concentration of the GTPs used among studies. The concentration of GTPs used in in vivo studies is routinely lower than that used in in vitro studies [105]. In one study, patients with prostate cancer received polyphenon E, which is derived from a hot water extract of green tea leaves (*Camellia sinensis*) and contains 85–95% catechins, including EGCG, for 3–6 weeks before surgery. However, GTP levels in the prostatectomy tissue were low to undetectable [106]. In addition, in a phase II randomized double-blind trial of bladder cancer, polyphenon E or placebo was administered prior to transurethral resection or radical cystectomy, following which tissue EGCG levels were compared between the experimental and placebo groups; there was no significant difference between the two groups [107]. On the basis of these findings, several investigators have shown interest in new technologies and methods that can achieve higher concentrations of GTPs in cancer tissues.

Nanoparticles are recognized as useful drug carriers. The use of EGCG–gold nanoparticles was found to be more effective than the use of free EGCG for treatment in an animal model of bladder cancer [67]. Moreover, EGCG–gold nanoparticles administered orally, intraperitoneally, and intratumorally suppressed tumor growth in C3H/He mice injected with murine bladder cancer cells (MBT-2). The anticancer effects were greater with intraperitoneal and intratumoral administration than with oral administration [67]. Another study showed that Mg(II)-catechin nanocomposite particles had anticancer effects in vitro and in vivo in a rat model of in situ bladder cancer [108]. In that study, Mg(II)-catechin nanocomposite particles that delivered siRNA targeting oncogene eukaryotic translation initiation factor 5A2, showed enhanced antitumor activity [108].

Various new treatment and prevention strategies based on GTPs have been developed in in vitro studies and animal experiments. Some of them have been confirmed to be effective in vivo in bladder cancer animal models. Therefore, there is now a need for translational studies of such in vitro and animal experiments in humans.

### 3.5. Safety

In general, GTPs are considered to be safe because they are natural compounds. GTPs were found to have no toxic effects on normal cells in the gallbladder [28]. Furthermore, 24-h treatment with 20 μg/mL and 40 μg/mL of EGCG increased the frequency of apoptotic cells in colon cancer cell lines (COLO205), while no significant changes were observed in normal colon epithelial cells (NCM460) [19]. Although green tea and its extracts have a relatively high level of safety for normal cells, it should be noted that “natural” does not always imply safety. In fact, a review on the toxicological effects of green tea showed that various side effects occurred in animal models, healthy volunteers, and patients, with hepatotoxicity, gastrointestinal disorders, and nervous system stimulation being the most important effects, although green tea and its main components are not recognized as major teratogenic, mutagenic, or carcinogenic substances [2]. The study also recommended that green tea should be used with caution in pregnancy, while breast feeding, and in susceptible individuals, as data in these contexts are limited [2].

In normal urothelial cells (UROtsa cell line), GTEs, including ECG and EGCG, were found to inhibit growth in a dose-dependent manner [62]. Another report mentioned that bladder cancer cells (SW780 cell line) were much more sensitive to EGCG than normal bladder epithelial cells (SV-HUC-1 cell line) [27]. In that study, 24-h treatment with EGCG at 100 mM induced cell proliferation inhibition of more than 70% in SW780 cells and of only 7.8% in SV-HUC-1 cells [27]. In addition to anticancer effects, preventive effects against oxidative stress in normal bladder cancer cells have been reported [78].

We previously discussed the anticancer effects of GTPs in a mouse model of BBN-induced bladder cancer [102,104]. When normal urothelial cells in mice treated with GTPs were examined by hematoxylin–eosin staining, abnormal morphological changes were not detected. However, more detailed analyses are necessary to confirm the safety and biological effects of GTPs on normal urothelial cells.

## 4. Future Direction

The anticancer effects of green tea in bladder cancer have been analyzed using comprehensive methods in epidemiological studies, in vitro studies using bladder cancer cell lines, animal models, and in vivo studies using human tissues. However, some limitations need to be overcome in order to understand the effects of green tea more accurately. Prospective randomized clinical trials with large study populations are yet to be performed to determine the anticancer effects of green tea in Western countries, including the USA, since epidemiological studies have provided some evidence that can serve as a foundation for such trials. On the other hand, such trials are difficult to design because the frequency and amount of green tea consumption in these countries are quite low.

As mentioned above, a combination of conventional chemotherapy and GTPs can be effective in several cancers, including bladder cancer [35,36,67]. In recent years, green tea has come to be known as a radiation sensitizer in prostate cancer [109]. However, there is little information on green tea-induced changes in terms of the anticancer effects of radiotherapy for bladder cancer. At present, immune checkpoint inhibitors are the major therapeutic agents used for patients with advanced/metastatic bladder cancer [110,111], and the usefulness of green tea as a sensitizer of immune checkpoint inhibitors has not yet been investigated. Therefore, more information on the efficacy and safety of combination therapy involving cytotoxic anticancer methods and green tea consumption or GTP intake in bladder cancer is warranted.

Clarification of the extent of the anticancer effects of green tea is difficult, because other bladder cancer-related external factors, such as smoking, exposure to chemical agents, and family history, also play a role [112,113,114,115]. In addition, the influence of other beverages, such as coffee, black tea, and oolong tea, and the total fluid intake should be investigated. Furthermore, GTP has complex molecular interactions that could contribute to its potential health benefits [80]. We hope that this review will encourage further investigations of the anticancer effects of green tea that could lead to the development of new treatment and prevention strategies based on green tea consumption and GTP intake, which are relatively safe.

## 5. Conclusions

In this review, we summarized studies on the potential anticancer effects of green tea consumption and GTP intake in bladder cancer by gathering data from epidemiological, in vivo, and in vitro studies, which have shown conflicting results. In vitro studies using cancer cell lines indicated that GTPs suppress malignant behavior via inhibition of cancer cell proliferation, migration, and invasion and inhibition of apoptosis, and some in vivo studies using animal model also showed similar results. However, some epidemiological studies showed no significant relationship between green tea consumption and the risk of bladder cancer. These discrepancies can be attributed to differences in the race, country, and measurement unit for green tea consumption and/or the influence of other factors, such as smoking, amount of green tea consumption per day, and the intake of other beverages. Therefore, more detailed and wider analyses of larger study populations from various countries, including USA, Europe, and Africa, are necessary to confirm the anticancer effects and usefulness of green tea as an anticancer agent. The anticancer effects of GTPs alone are considered to be limited, whereas a combination of GTPs and other strategies, such as chemotherapy, radiotherapy, immune therapy, and molecular targeted therapy is expected to have some clinical benefit in patients with cancer, including bladder cancer.

## Figures and Tables

**Table 1:** Previous reports on the relationship between green tea consumption and bladder cancer risk.

Case-Control Study				Cohort Study			
Author/Year /Country/Ref	Daily Intake	OR/RR	95% CI	Author/Year Country/Ref	Daily Intake	OR/RR	95% CI
Waikens et al.	Cases/controls = 271/522			Chyou et al.			
/1996	(Men)			/1993	Never	1.0	
/USA	Q1	1.0		/USA	Ever	1.34	0.79–2.27
/[49]	Q2	1.1	0.6−1.9	/[50]			
	Q3	1.1	0.6–2.3				
	(Women)			Nagano et al.			
	Q1	1.0		/2000	0–1 cup	1.0	
	Q2	0.8	0.3–2.1	/Japan	2–4	1.07	0.61–2.00
	Q2	0.9	0.3–2.6	/[51]	≥5	1.07	0.58–2.08
Wakai et al.	124/744			Kurahashi et al.			
/2004	<	1.0		/2009	(Men)		
/Japan	1–4	1.40	0.74–2.62	/Japan	<1 cup	1.0	
/[47]	5–9	2.67	1.44–4.94	/[52]	1–2	1.18	0.73–1.91
	≥10	1.18	0.49–2.84		3–4	0.71	0.43–1.18
					≥5	0.90	0.56–1.45
Hemelt et al.	381/371				(Women)		
/2010	0	1.00			<3	1.0	
/China	<Daily	0.83	0.54–1.27		3–4	1.22	0.49–3.00
/[53]	Daily	1.02	0.71–1.48		≥5	2.29	1.06–4.92
	<4	1.23	0.76–1.97				
	≥4	0.83	0.53–1.28				
Wang et al.	1007/1299						
/2013	Never	1.0					
/USA	0.1–0.13	0.82	0.61–1.11				
/[49]	≥0.14	0.60	0.45–0.79				

ref: reference. OR: odds ratio. RR: relative ratio. CI: confidential interval.

**Table 2:** Meta-analyses of the relationship between green tea consumption and bladder cancer risk.

Author/Year /Reference	Number of Case-Control Studies	Number of Cohort Studies	Odds Ratio	95% Confidence Interval
Qin et al./2012/[55]	2	3	0.97	0.73–1.21
Wang et al./2013/[13]	2	2	0.81	0.68–0.98
Wu et al./2013/[56]	3	2	1.03	0.82–1.31
Zhang et al./2015/[57]	0	3	1.02	0.95–1.11
Weng et al./2017/[58]	4	3	0.95	0.73–1.24

**Table 3:** Relationships between green tea polyphenol treatment and various cancer-related functions in in vitro studies of bladder cancer cell lines.

Cell Line	Type	Dosage/Concentration	Author/Year/Reference
Growth inhibition			
AY-27	EGCG	>100 μM	Kemberling et al./2003/[61]
NBT-II	EGCG	10, 20, or 40 μM/L	Chen et al./2004/[62]
J82	EGCG	70–87 μM	Rieger et al./2007/[63]
UM-UC-3	EGCG	70–87 μM	Rieger et al./2007/[63]
EJ	EGCG	70–87 μM	Rieger et al./2007/[63]
T24	EGCG	70–87 μM 20–100 μg/mL 20–80 μg/mL	Rieger et al./2007/[63]; Qin et al./2007/[64]; Philips et al./2009/[65]
KK47	EGCG	70–87 μM	Rieger et al./2007/[63]
TCCSUP	EGCG	70–87 μM 10–80 μg/mL	Rieger et al./2007/[63]; Philips et al./2009/[65]
TSGH-8301	EGCG	25–100 μM	Chen et al./2011/[66]
MBT-2	EGCG	12.5–50 μM	Hsieh et al./2011/[67]
RT4	EGCG	60–80 μg/mL	Philips et al./2009/[65]
SW780	EGCG	10–80 μg/mL	Philips et al./2009/[65]
Apoptosis induction			
NBT-II	EGCG	10 μM/L	Chen et al./2004/[62]
T24	EGCG	10–80 μg/mL	Qin et al./2007/[64]
TCCSUP	EGCG	40 μg/mL	Philips et al./2009/[65]
TSGH-8301	EGCG	75 μM	Chen et al./2011/[66]
MBT-2	EGCG	50 μM	Hsieh et al./2011/[67]
SW780	EGCG	50–200 μM	Luo et al./2017/[27]
Migration inhibition			
UM-UC-3	EGCG	5 μM	Rieger et al./2007/[63]
EJ	EGCG	5 μM	Rieger et al./2007/[63]
TCCSUP	EGCG	5 μM	Rieger et al./2007/[63]
SW780	EGCG	25–50 μM	Luo et al./2017/[27]
Cell cycle arrest			
NBT-II	EGCG	10, 20, or 40 μM/L	Chen et al./2004/[62]

EGCG: epigallocatechin-3-gallate. GTP: green tea polyphenol.

**Table 4:** Potential molecular targets of green tea polyphenols in bladder cancer cell lines.

Molecules	Cell Line	Author/Year/Reference
Bcl-2 family	T24	Qin et al./2007/61; Gu et al./2017/[68]
	TSGH-8301	Chen et al./2011/[66]
	MBT-2	Hsieh et al./2011/[67]
	SW780	Luo et al./2017/[27]
	BIU87	Gu et al./2017/[68]
Caspase family	TSGH-8301	Chen et al./2011/[66]
	MBT-2	Hsieh et al./2011/[67]
	SW780	Luo et al./2017/[27]
Cyclin D1	NBT-II	Chen et al./2004/[62]
Cyclin-dependent kinase 4/6	NBT-II	Chen et al./2004/[62]
Heat shock protein 27	TSGH-8301	Chen et al./2011/[65]
JNK/Bcl-2/Beclin-1	T24, BIU87	Gu et al./2017/[75]
Matrix metalloproteinase-9	SW780	Luo et al./2017/[27]
N-cadherin	UM-UC-3	Rieger et al./2007/[63]
Nuclear factor-kappa B	TCCSUP	Philips et al./2009/[65]
	SW780	Luo et al./2017/[27]
Phosphatidylinositol 3-kinase	UM-UC-3	Rieger et al./2007/[63]
/Akt signaling	T24	Qin et al./2007/[64]
	TSGH-8301	Chen et al./2011/[66]
Retinoblastoma protein	NBT-II	Chen et al./2004/[62]
Wnt signaling	TCCSUP	Philips et al./2009/[65]

**Table 5:** Anticancer effects of green tea in animal models of bladder cancer.

Author/Year/Reference	Agents	Methods	Animal Model	Summary of Results
Sato et al./1999/ [100]	GTE	Drinking	Rat; Chemically induced	Dose-dependently inhibited tumor growth when administered after the carcinogen
Sato et al./2003/ [101]	GTE	Drinking	Rat; Chemically induced	Prevented tumor growth when administered before the carcinogen
Kemberling et al./2003/[61]	EGCG	Intra-vesical	Rat; intravesical implantation	Inhibited the growth of transitional carcinoma cells
Rieger et al./2007/[63]	EGCG	Drinking	Xenograft model	Over 50% decrease in the mean final tumor volume
Sagara et al./2010/[102]	GTE	Drinking	Mouse; Chemically induced	Inhibited tumor growth and invasion via regulation of angiogenesis
Chen et al./2011/[66]	ECGC	Gavage	Mouse; xenograft model	Inhibited tumor growth in a dose-dependent manner
Hsieh et al./2011/[67]	EGCG	Orally, intraperitoneally, intratumor	Mouse; injection of cancer cells	EGCG–gold nanoparticles were more effective than free EGCG in inhibiting tumor growth
Henriques et al./2014/[103]	Whole green tea	Drinking	Mouse; Chemically induced	Influenced inflammation in the urothelium, but not carcinogenesis
Matsuo et al./2017/[104]	GTE	Drinking	Mouse; Chemically induced	Inhibited tumor growth and angiogenesis via human-antigen R-related pathways

ref: reference. GTEs: green tea extracts. ECGC: epigallocatechin-3-gallate.
